# Thrombospondin-1 promotes cell migration, invasion and lung metastasis of osteosarcoma through FAK dependent pathway

**DOI:** 10.18632/oncotarget.17427

**Published:** 2017-04-26

**Authors:** Chuanzhen Hu, Junxiang Wen, Liangzhi Gong, Xu Chen, Jun Wang, Fangqiong Hu, Qi Zhou, Jing Liang, Li Wei, Yuhui Shen, Weibin Zhang

**Affiliations:** ^1^ Department of Orthopaedics, Ruijin Hospital, Shanghai Jiaotong University School of Medicine, Shanghai 200025, People's Republic of China; ^2^ Shanghai Key Laboratory for Bone and Joint Diseases, Shanghai Institute of Orthopedics and Traumatology, Ruijin Hospital, Shanghai Jiaotong University School of Medicine, Shanghai 200025, People's Republic of China; ^3^ Wuxi Xinrui Hospital, Department of Orthopaedics, Wuxi Branch, Ruijin Hospital, School of Medicine, Shanghai Jiaotong University, Wuxi 214028, People's Republic of China

**Keywords:** thrombospondin-1, osoteosarcoma, lung metastasis, FAK

## Abstract

Microenvironment at the metastatic locus usually differs greatly from that present in the site of primary tumor formation and it has a significant impact on the fate of the extravasated cancer cells. We compared gene expression signatures of primary tumors and lung metastatic tumors, and identified Thrombospondin-1 (TSP1) as highly up-regulated in the lung metastatic tumors. Immunohistochemical staining further indicated that TSP1 protein expression was higher in lung metastatic tumors compared to primary tumors in both osteosarcoma xenograft model and human clinical samples. TSP1 mRNA level is significantly associated with the Enneking stage of osteosarcoma and lung metastasis. TGF-β pathways could stimulate the TSP1 expression in osteosarcoma cells. Knockdown of TSP1 expression in osteosarcoma cells dramatically suppressed cell wound healing, migration and invasion. Treatment with recombinant TSP1 protein in osteosarcoma cells significantly promoted cell wound healing, migration and invasion. Meanwhile, suppression of TSP1 in osteosarcoma cells resulted in decreased pulmonary metastasis *in vivo*. Mechanistically, TSP1 increased expression of metastasis related genes, including MMP2, MMP9 and Fibronectin 1. TSP1 promoted osteosarcoma cell motility through the activation of FAK pathway. Taken together, our study provides evidence of the contributions of TSP1 to the lung metastasis of osteosarcoma and suggests that this protein may represent a potential therapeutic target for osteosarcoma lung metastasis.

## INTRODUCTION

Tumor metastasis is still the main cause of cancer-related mortality, so it is important to identify the key molecules in each step of tumor metastasis and develop new strategies for prevention and control of tumor metastasis [1–3]. A key step in metastasis is that the extravasated carcinoma cells must survive in the foreign microenvironment and ultimately develop into macroscopic metastases. However, the microenvironment at the metastatic locus usually differs greatly from that present in the site of primary tumor formation and it has a significant impact on the fate of the extravasated cancer cells [4, 5].

Thrombospondin-1 (TSP1) is an important matri-cellular glycoprotein in tumor microenvironment [6]. Because of its structure with different structural and functional domains, it has a variety of functions [7, 8]. Previously, TSP1 has been reported as a major endogenous inhibitor of angiogenesis and its role of inhibiting tumor progression has been well documented [9, 10], however, its role in tumor metastasis is only just emerging. TSP1 induced cell migration in several tumor cell lines, suggesting that TSP1 assists the cancer invasion [11–13].

We previously performed a microarray to compare the different gene expression between metastatic tumor and primary tumor in the osteosarcoma orthotopic lung metastasis model in mice. Surprisingly, we found that TSP1, a major endogenous inhibitor of angiogenesis, was highly expressed in metastatic tumors than in primary tumors. Therefore, we assumed that TSP1 plays a role in promoting lung metastasis of osteosarcoma. This study analyzed whether TSP1 highly expressed in patients’ samples and TSP1 promoted the migratory, invasive, and metastatic phenotype of osteosarcoma.

## RESULTS

### TSP1 expression is acquired in the malignant progression of osteosarcoma

**Figure 1:**
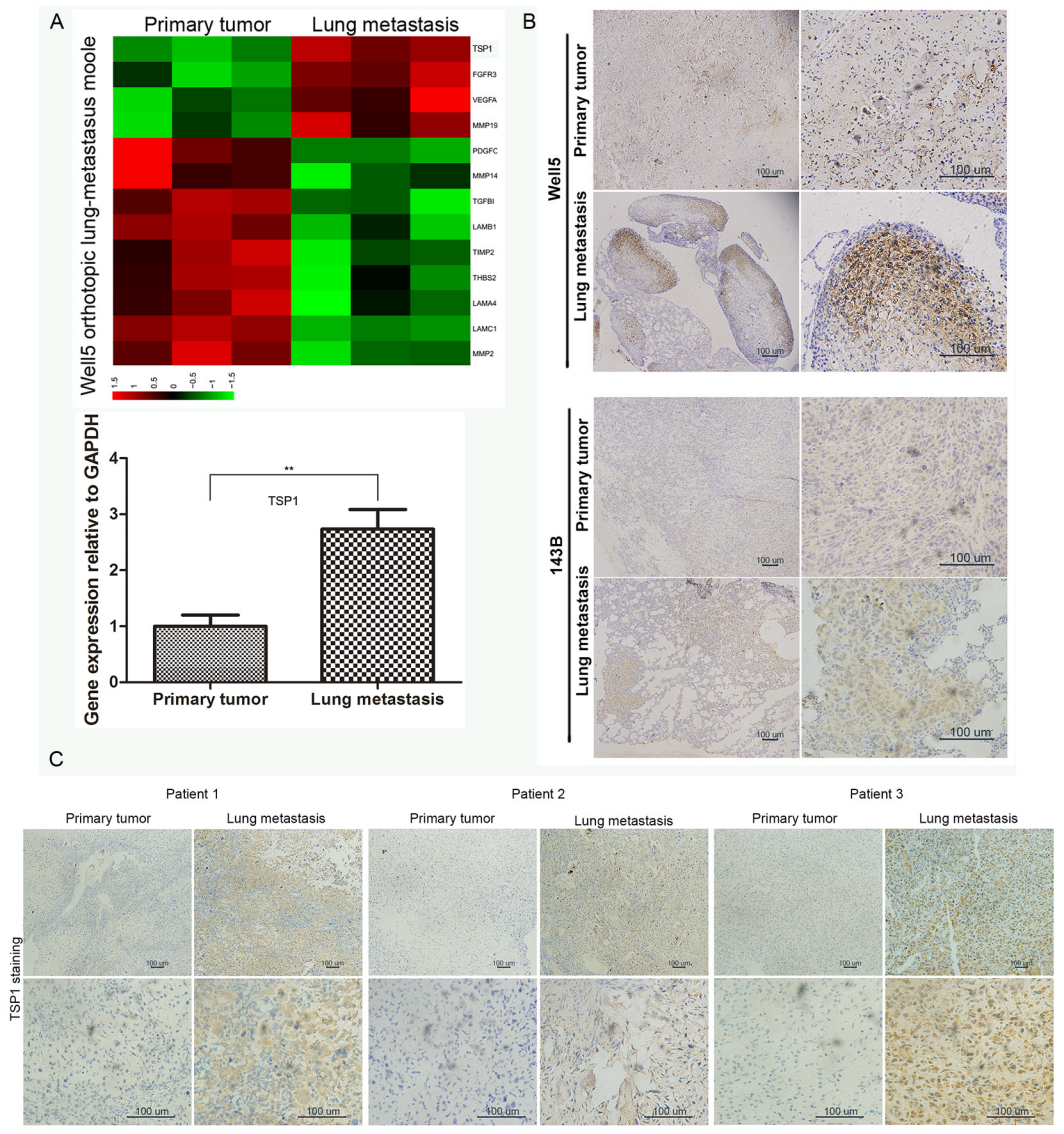
Expression of TSP1 in osteosarcoma tissues **(A)** Primary tumor cells and lung metastatic tumor cells freshly isolated from Well5-derived orthotopic lung metastasis model and its RNA was extracted and analyzed by microarray. Upper panel, the differentially expressed angiogenesis-related genes were selected for heatmap display. Bottom panel, Real-time quantitative PCR was performed to detect TSP1 expression in the expression profile. **P<0.01. **(B)** Immunohistochemical staining of TSP1 protein level in primary tumor and lung metastasis from Well5 and another osteosarcoma cell line 143B derived orthotopic lung metastasis model. **(C)** Immunohistochemical staining of TSP1 in primary and metastatic tumor samples of three osteosarcoma patients.

In order to find differences between gene expression of metastatic tumor cells and primary tumor cells, we collected freshly isolated primary tumor cells and lung metastatic tumor cells, from human osteosarcoma cell line Well5-derived orthotopic lung metastasis model in mice, for microarray expression analysis. Interestingly, in differentially expressed angiogenesis-related genes, thrombospondin-1 (TSP1), an anti-angiogenic gene, was higher expression in metastatic tumor cells than in primary tumor cells (Figure 1A). qRT-PCR also confirmed this difference (Figure 1A). To further confirm this finding, TSP1 protein expression was analyzed in Well5 and another osteosarcoma cell line 143B derived orthotopic lung metastasis model (Figure 1B). TSP1 staining was particularly evident at the invasive front of lung metastatic nodules, whereas it had no expression in normal lung tissue and was low in primary tumor. In addition, TSP1 protein expression was analyzed in human osteosarcoma samples by Immunohistochemical staining. We found that TSP1 staining was particularly evident in lung metastasis, whereas it was low in primary tumor (Figure 1C).

We next investigated the TSP1 mRNA level in 40 frozen surgical biopsy specimens from patients with clinically osteosarcoma who did not receive any prior chemotherapy or radiation therapy before surgery. TSP1 mRNA level is significantly associated with the Enneking stage of osteosarcoma and lung metastasis, whereas there was no significant association between TSP1 mRNA level and patients’ age, sex, tumor size, or local recurrence (Table 1). The Enneking system is based on the histological grade of the tumor, its local extent and the presence or absence of metastasis [14]. As compared with stage II A and stage II B osteosarcoma, there was a significant increase of TSP1 mRNA expression in stage III tumors, which presented with distant metastasis. Furthermore, TSP1 mRNA level was significant higher in samples with lung metastasis (P<0.0008), suggesting TSP1 might be involved in the distant metastatic process of osteosarcoma.

**Table 1:** Association between TSP1 mRNA level in primary tumor samples and the clinic-pathological characteristics of osteosarcoma patients

Characteristics	n	TSP1 mRNA Mean (range)	P
All patients	40	0.702 (0.01-3.42)	
Age			
<18	22	0.595 (0.01-1.56)	
≥18	18	0.833 (0.01-3.42)	0.274
Gender			
Male	25	0.586 (0.01-1.56)	
Female	15	0.900 (0.01-3.42)	0.161
Clinical stage			
II A	12	0.485 (0.01-1.32)	0.361
II B	20	0.620 (0.01-1.56)	**0.015**
III	8	1.413 (0.15-3.42)	**0.012**
Tumor site			0.702
Femur	19	0.8 (0.01-2.08)	
Tibia or fibula	15	0.615 (0.24-3.42)	
Other	6	0.798 (0.15-1.28)	
Tumor size			
<10 cm	22	0.588 (0.01-2.08)	
≥10 cm	18	0.861 (0.12-3.42)	0.184
Local recurrence			
NO	26	0.946 (0.01-3.42)	
Yes	14	0.571 (0.01-2.08)	0.094
Lung metastasis			
NO	27	0.4667 (0.01-1.21)	
Yes	13	1.192 (0.08-3.42)	**0.0008**

### TGF-β stimulated TSP1 expression in osteosarcoma cells

**Figure 2:**
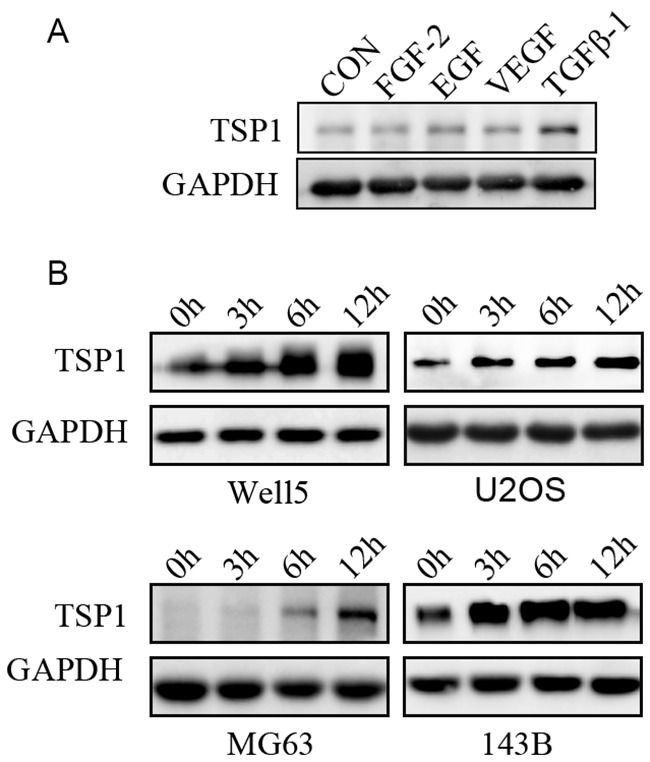
Regulation of TSP1 expression in osteosarcoma cells by TGF-β pathways **(A)** Western blotting assays for the levels of TSP1 in Well5 cells treated with PBS, FGF-2(10 ng/ml), EGF (20 ng/ml), VEGF (5 ng/ml), TGF-β1(5 ng/ml) for 12h. GAPDH was used as a loading control. **(B)** Western blotting assays for the levels of TSP1 in Well5, U2OS, MG63and 143B cells treated with 5 ng/ml TGF-β1 for the indicated time points. GAPDH was used as a loading control.

We next thought the mechanisms inducing TSP1 expression in osteosarcoma tumor cells. Several cytokines, including FGF-2, VEGF, EGF, TGF-β, have been reported to regulate the expression of TSP1 [15–17]. We thus measured the expression of TSP1 in Well5 cells stimulated with FGF-2 or VEGF or EGF or TGF-β1. We observed that TGF-β1 stimulated TSP1 production inWell5 cells whereas FGF-2, VEGF, and EGF did not (Figure 2A). To further assess the production of TSP1 by TGF-β1 in osteosarcoma cells, four osteosarcoma cell lines, WELL5, U2OS, MG63 and143B, were stimulated with 5 ng/ml TGF-β1 at different time points. Western blot analysis confirmed the induction of TSP1 by TGF-β1, showing a time-dependent increase of TSP1 expression in four osteosarcoma cell lines (Figure 2B). These results suggest that TGF-β pathway regulates the expression of TSP1 in osteosarcoma.

### TSP1 accelerates wound healing of osteosarcoma cells

**Figure 3:**
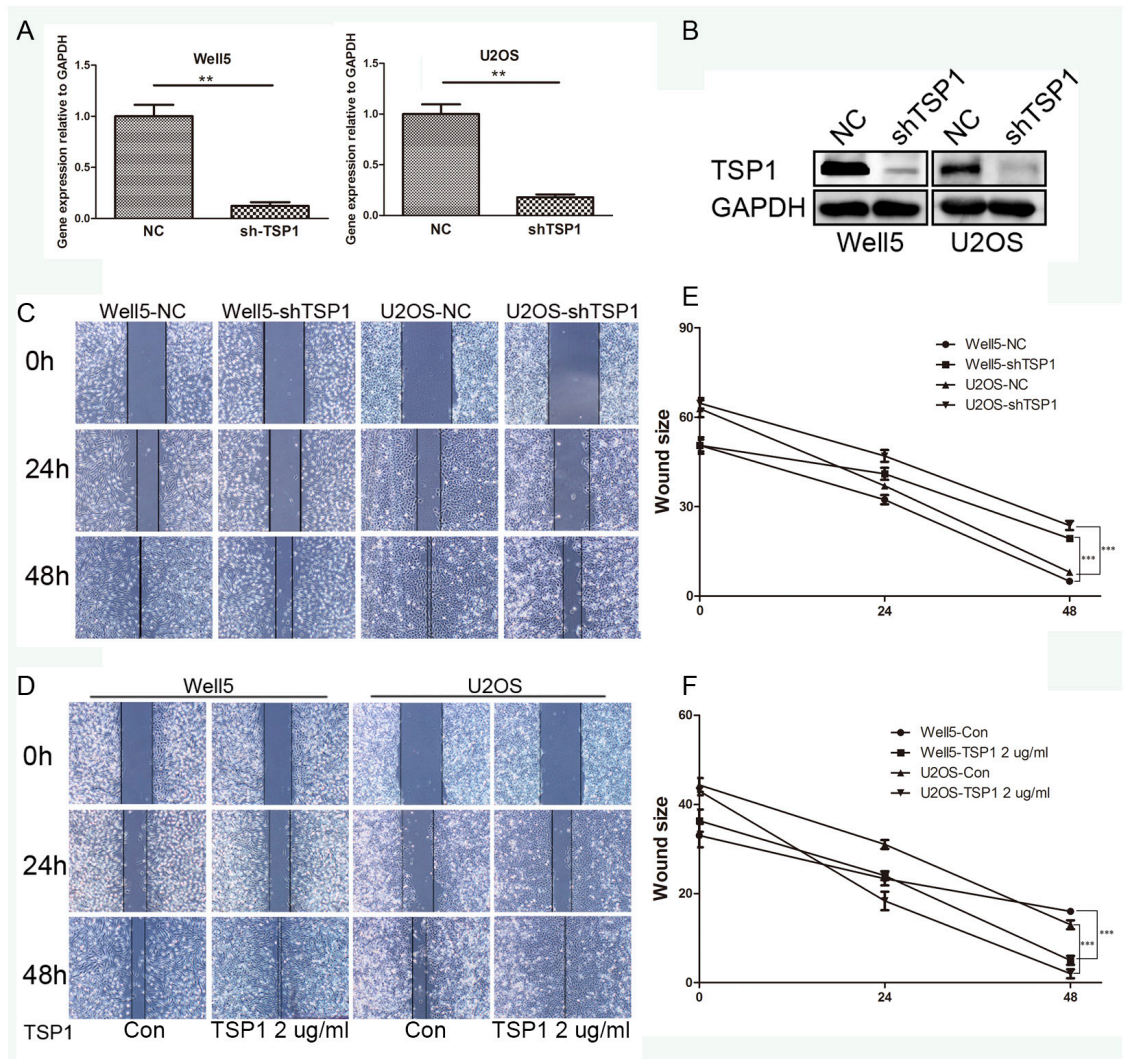
Effects of TSP1 on wound healing in osteosarcoma cells **(A)** The expression of TSP1 in Well5 and U2OS cell lines transfected with negative control (NC) or lenti-shTSP1 was evaluated by qRT-PCR. Data from 3 independent experiments were expressed as the mean ± S.D. **P<0.01. **(B)** Decreased protein level of TSP1 was revealed by western blotting after transfection with lenti-shTSP1, consistent with that of qRT-PCR. GAPDH was used as a loading control. **(C)** Wound healing assay using lenti-TSP1 and negative control transfected Well5 and U2OS cells. **(D)** Wound healing assay using Well5 and U2OS cells treated with PBS (control) or TSP1 2 ug/ml. Microscopic observations were recorded 0, 24 and 48 hours after scratching the cell surface. A representative image from every independent experiment is shown. **(E, F)** The distances between wound edges of osteosarcoma cells at 0, 24 and 48 hours. ***P<0.001

To study the function of TSP1 in osteosarcoma, we firstly confirmed the expression of TSP1 protein in osteosarcoma cell line by western blot. TSP1 protein expression was much higher in Well5 and U2OS cells than in 143B and MG63 cells (Supplementary Figure 1A). Thus Well5 and U2OS cells were infected with lentivirus containing shRNA constructs against TSP1 (shTSP1) or negative control scrambled shRNA (NC). As shown in Figure 3A and 3B, shTSP1 markedly decreased TSP1 expression in both osteosarcoma cells compared to NC-transfected cells, with no effect on cell proliferation or apoptosis (Supplementary Figure 1B and 1C).

To investigate the effect of TSP1 on movement ability and wound healing, we performed a wound healing assay in which we observed longer distance in wound healing of Well5-shTSP1 and U2OS-shTSP1 cells compared with negative control groups (Figure 3C and 3E). Additionally, distance was markedly shorter in WELL5 and U2OS cells with human recombinant TSP1 protein treatment than those of negative control cells (Figure 3D and 3F).

### TSP1 promotes the migration and invasion of osteosarcoma cells

**Figure 4:**
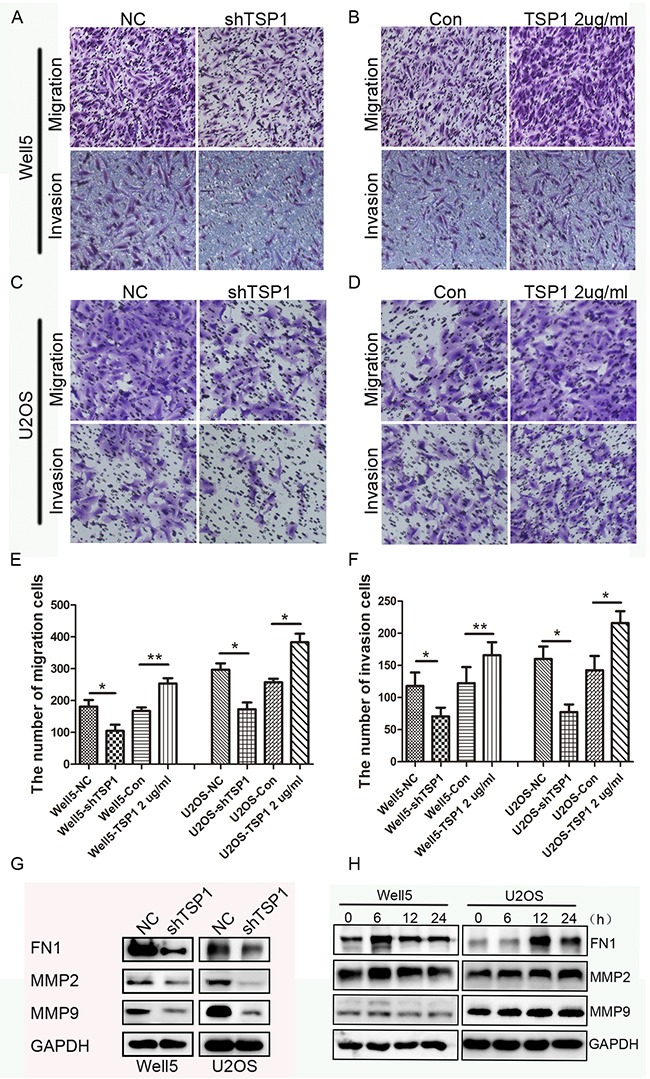
Effects of TSP1 on migration, invasion ability in osteosarcoma cells **(A, C)** Trans-well migration and invasion assay using lenti-TSP1 and negative control transfected Well5 and U2OS cells. **(B, D)** Trans-well migration and invasion assay using Well5 and U2OS cells treated with PBS (control) or TSP1 2 ug/ml. **(E, F)** Bar graph of trans-well migration and invasion assay representing the mean value ± SD from independent experiments performed in triplicate. *P < 0.05; **P < 0.01. **(G)** Western blotting analysis of MMP2, MMP9 and FN1 expression in lenti-TSP1 and negative control transfected Well5 and U2OS cells. **(H)** Western blotting analysis of MMP2, MMP9 and FN1expression in Well5 and U2OS cells treated with TSP1 2 ug/ml for different time point.

Next, we determined TSP1 action on migration and invasion using a Boyden chamber assay and found that Well5-shTSP1 and U2OS-shTSP1 cells exhibited reduced migration rate and invasion rate relative to their respective control cells (Figure 4A, 4C, 4E and 4F). Then we further confirmed the effects of TSP1 on osteosarcoma cell migration and invasion by using recombinant human TSP1 protein. The results showed that recombinant TSP1 protein markedly promoted Well5 and U2OS cell migration and invasion (Figure 4B, 4D, 4E and 4F). Collectively, these data above suggest that TSP1 can promote metastatic characteristics *in vitro* in OS cell lines.

Next, we examined the effect of TSP1on the molecules that associate with tumor metastasis. MMP2, MMP9 and FN1 (Fibronectin 1) play important roles in tumor metastasis and are closely correlated with the migration and invasion of tumor cells [18–21]. We found that MMP2, MMP9 and FN1 were significantly decreased in WELL5-Sh-TSP1 and U2OS-Sh-TSP1 cells compared to negative control cells (Figure 4G), while human recombinant TSP1 protein could stimulate the expression of MMP2, MMP9 and FN1 in Well5 and U2OS cells and reached peak at 6h and 12h, respectively (Figure 4H). These results suggest that TSP1 promotes osteosarcoma cell migration and invasion through stimulating the expression of MMPs and FN1.

### TSP1 promotes osteosarcoma cell migration and invasion through the activation of FAK pathway

**Figure 5:**
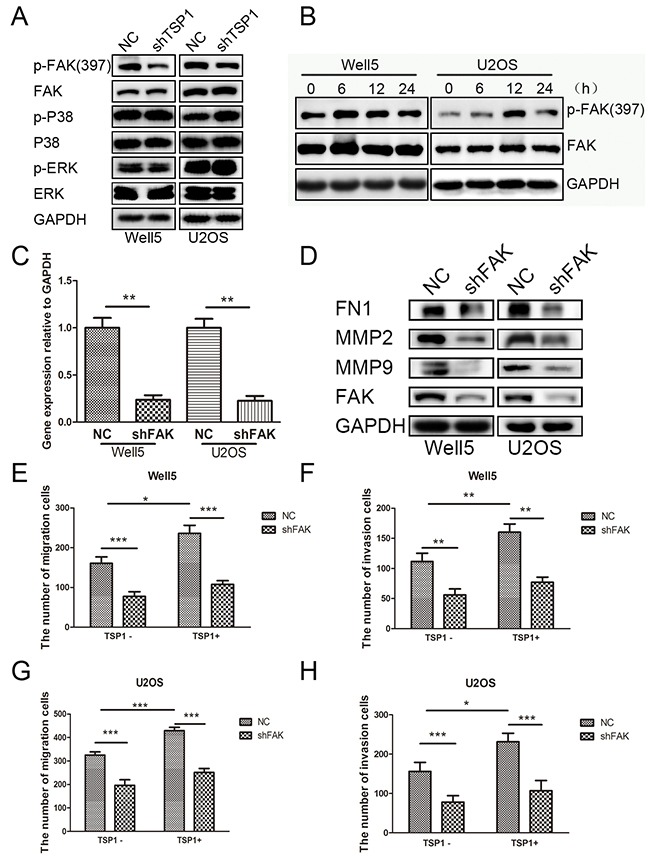
TSP1 promotes osteosarcoma cell migration and invasion through the activation of FAK pathway **(A)** Western blotting analysis of ERK, P38MAPK, FAK phosphorylation in lenti-TSP1 and negative control transfected Well5 and U2OS cells. **(B)** Western blotting analysis of FAK phosphorylation in in Well5 and U2OS cells treated with TSP1 2ug/ml for different time point. **(C)** Knockdown efficiency of FAK in Well5 cells and U2OS cells as demonstrated by qRT-PCR. **P < 0.01. **(D)**. Western blot detected the MMP2, MMP9 and FN1 expression in Well5 and U2OS cells after FAK were silenced by siRNAs. **(E-H)** Trans-well migration and invasion assay in lenti-shFAK and negative control transfected Well5 and U2OS cells treated with PBS or TSP1 2 ug/ml. Bar graph representing the mean value ± SD from independent experiments performed in triplicate. *P < 0.05; **P < 0.01; ***P < 0.001.

Next, we investigated the downstream pathway of TSP1 in osteosarcoma cells. Previous study reported that TSP1 regulated cell migration through ERK, P38MAPK or FAK pathway in several diseases [16, 22–25]. Thus, we examined whether TSP1 mediated cell migration and invasion of osteosarcoma through ERK, P38MAPK or FAK pathway. As show in Figure 5A, knockdown of TSP1 resulted in decreased phosphorylation levels of FAK in WELL5 and U2OS cells, whereas there was no significant change in phosphorylation levels of both ERK and P38. In addition, TSP1 protein treatment remarkably promoted the phosphorylation levels of FAK in Well5 and U2OS cells and reached peak at 6h and 12h, respectively (Figure 5B). To further confirm the role of FAK signaling in TSP1-induced osteosarcoma cell migration and invasion, we detected the MMP2, MMP9 and FN1 expression in Well5 and U2OS cells after FAK were silenced by siRNAs (Figure 5C). MMP2, MMP9 and FN1 expression were markedly decreased after FAK was knock-down in Well5 and U2OS cells Figure 5D. In addition, the enhanced effects of TSP1 on osteosarcoma cell migration and invasion were completely blocked by the silencing of FAK in both Well5 (Figure 5E and 5F) and U2OS (Figure 5G and 5H) cells. Collectively, these results indicate that TSP1 promotes osteosarcoma cell migration and invasion through the activation of FAK pathway.

### Knockdown of TSP1 inhibited lung metastasis of osteosarcoma cells *in vivo*

**Figure 6:**
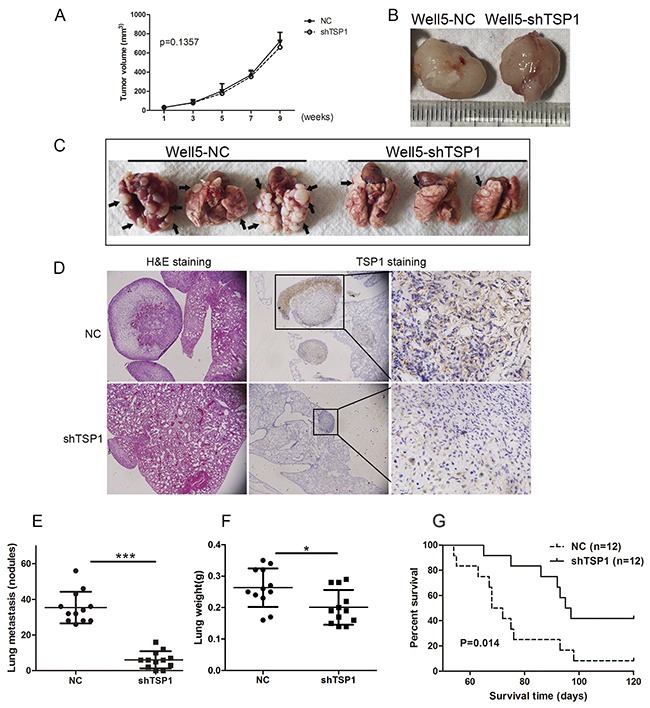
Sliencing of TSP1 inhibits lung metastasis of osteosarcoma cells **(A)** Knockdown of TSP1 did not inhibit primary tumor growth of osteosarcoma *in vivo* (P>0.05). **(B)** Two representative tissue samples retrieved from primary tumors formed by negative control and lenti-shTSP1 transfected Well5 cells in xenograft, respectively. **(C)** Macroscopic appearances of lung metastasis are shown. The black arrows indicate macroscopic pulmonary metastatic lesions. **(D)** Representative hematoxylin-eosin stained images (left panel) and IHC staining images (middle and right panel) of the lungs shown in BC **(E)** Number of tumor nodules was evaluated and analyzed between Well5 cells transfected with lenti-shTSP1 and negative control group. ***P< 0.001. **(F)** Mean weight of the lungs. *P< 0.05. All values in D and E are mean+ SD of the 12 mice in negative control group and shTSP1 group, respectively. **(G)** TSP1 silencing led to later death of mice seen by survival curve analysis. *P< 0.05, n= 12.

Based on the *in vitro* findings described above, we next determined the effects of TSP1 on tumor metastasis *in vivo*. As shown in Figure 6A and 6B, stable suppression of TSP1 in Well5 cells did not affect primary tumor growth in xenograft. However, TSP1 knockdown considerably inhibited lung metastasis of osteosarcoma cells, as reflected by the number and weight of the metastatic nodules (Figure 6C–6F). As show in the middle and right panel of Figure 6C, TSP1 expression in metastatic nodules of shTSP1 group was completely inhibited compared to negative control group. In addition, silencing of TSP1 also led to later death of mice (Figure 6G).

## DISCUSSION

This study reports that TSP1 expression is highly expressed in osteosarcoma lung metastasis than in primary tumors and increased expression of TSP1 is associated with an invasive and metastatic phenotype of osteosarcoma.

Previously, TSP1 was reported to be an inhibitor of angiogenesis and tumor progression and its expression was lost in many malignant tumors [10, 26, 27]. Recently, however, a contradictory conclusion, that TSP1 promote tumor migration, invasion and metastatic to distal organ in breast cancer, thyroid cancer, prostate cancer and melanoma, is drawn [12, 13, 15, 28, 29]. Lawler reported that fewer osteosarcomas occur in p53-deficient mice that lack TSP1 as compared to those that express TSP1 [30]. In our study, we firstly report that TSP1 expression was significantly higher in osteosarcoma metastatic nodules than in primary tumors, and its expression in clinical samples was associated with Enneking stage of osteosarcoma and lung metastasis. These observations indicate that TSP1 expression acquired during metastatic progression of osteosarcoma.

In some tumor cells, a positive feedback loop betweenTSP1 and TGFβ may exist in that TSP1 is an activator of TGFβ and active TGFβ induces TSP1expression [31–33]. In our study, TSP1 expression was stimulated in osteosarcoma cell lines by TGF-β1, not by other growth factors (VEGF, EGF, FGF-2), and this is accordance with previous study that TGF-β1 not EGF could stimulate TSP1 expression in osteosarcoma cell line MG63 [34]. Masahiro et al. [34] reported that treatment of human osteosarcoma cell line MG63 with TGF-β1 induced enhanced expression of TSP1 mainly through stabilization of its mRNA and P38MAPK pathway involved in TGF-β1-induced up-regulation of TSP1 mRNA. TGF-β1 is one of the critical growth factors that secreted by osteosarcomas and its expression is associated with high-grade osteosarcoma and lung metastasis [35–37]. Thus, we assume that a positive feedback loop between TSP1 and TGFβ may exist in osteosarcoma and this positive feedback loop may contribute to a more aggressive phenotype of osteosarcoma.

Next, we examine whether TSP1 expression is associated with a more aggressive phenotype of osteosarcoma *in vitro* and *in vivo*. Thus we performed knockdown assay to evaluate the implications of TSP1 on the metastatic ability of cell wound healing, migration and invasion. We found that metastatic behavior in Well5 and U2OS cells was remarkably inhibited by silencing TPS1 both *in vitro*, while Well5 and U2OS cells treated with TSP1 protein, ability of cell wound healing, migration and invasion were significantly promoted. Consistent with these results, the expression of several metastasis-related protein including MMP2, MMP9 and Fibronection 1 significantly decreased in Well5 and U2OS cells with TSP1 knockdown and increased in Well5 and U2OS cells treated with TSP1 protein. Silencing TSP1 expression was able to inhibit lung metastasis without affection to primary tumor growth in Well5-derived osteosarcoma orthotopic lung metastasis model in mice. Based on these results, we speculated that TSP1 might function as a modulator in the metastatic potential of osteosarcoma cells. Our results was consist with other studies reported that TSP1 promotes cell invasion in breast cancer, thyroid cancer, colon cancer, melanoma and prostate tumors [13, 15, 28, 29, 38]. In addition, knock-out of TSP1 in an animal model of breast cancer led to growth of the primary tumor but a decrease in the number of metastases [39].

Several signal pathways have been reported to involve in the cell migration regulated by TSP1, including ERK, P38MAPK, and FAK pathway [16, 23–25, 38]. In our experiment, we find that TSP1 knockdown have no effects on both ERK and P38 pathway, while FAK pathway was inhibited in TSP1 knockdown osteosarcoma cells and activated in osteosarcoma cells treated with TSP1 protein. Furthermore, FAK-specific siRNA was able to reverse the increased migration and invasion ability stimulated by TSP1protein in osteosarcoma cells. To date, most evidence suggests that FAK overexpression and FAK phosphorylation are markers for invasive and metastatic tumors, including Gastric cancer, colon cancer, thyroid cancer, ovarian cancer, prostate cancer, oral cancer [40–45]. Ren found that FAK overexpression and phosphorylation was associated with more aggressive osteosarcoma and siRNA-based knockdown of FAK dramatically reduced the migration and invasion of osteosarcoma cell line MG63 and 143B [46]. This, together with results of the current study, suggests that TSP1 may promote osteosarcoma cell migration and invasion through FAK pathway.

To best of our knowledge, this is the first study that systemically determines the expression pattern and cellular functions of TSP1 in osteosarcoma. Our data provides evidence that TGF-β1 increases TSP1 expression in progression of osteosarcoma lung metastasis and higher expression of TSP1 in osteosarcoma contribute to the lung metastasis through FAK pathway. Thus, TSP1 may serve as a target in strategies to better treatment of malignant osteosarcoma.

## MATERIALS AND METHODS

### Cell lines and cell culture

Osteosarcoma cell line 143B, MG63, U2OS were purchased from American Type Culture Collection (Manassas, VA, USA). Well5 was established by our group [47]. All cells were cultured in Dulbecco's Modified Eagle Medium (DMEM; Invitrogen), supplemented with 10% fetal bovine serum (FBS, Invitrogen), penicillin (100 U/ml) and streptomycin (100 mg/ml; Invitrogen), at 37°C in a 5% CO2 incubator.

For continuous exposure to human TSP1 (R&D Systems) or human FGF-2 (R&D Systems) or human VEGF (Peprotech) or human EGF (R&D Systems) or human TGF-β1 (Peprotech), cells were seeded on culture dishes that had been coated with TSP1 (2 ug/ml) or FGF-2(10 ng/ml) or EGF (20 ng/ml) or VEGF (5 ng/ml) or TGF-β1(5 ng/ml) for 12h.

### Clinical samples and data

Forty patients with osteosarcoma underwent surgical biopsy with no prior chemotherapy or radiation therapy in Ruijin hospital in Shanghai, China from January 2010 to December 2013. All patients provided written informed consent for the use of their tumor samples under a protocol approved by the ethics committee of Shanghai Ruijin Hospital. Two pathologists respectively reviewed all of the cases.

### TSP1 and FAK knock-down cells construction

The shRNA of TSP1, FAK and non-target control shRNA were purchased from Shanghai Genechem Co., Ltd, to target the following cDNA sequences: shTSP1, 5’-GCGUGUUUGACAUCUUUGATT-3’; shFAK, 5’-GGUUCAAGCUGGAUUAUUU-3; non-target control shRNA, 5′-TTCTCCGAACGTGTCACGT-3’.

### Gene Expression Profiling

Metastatic tumor cells and primary tumor cells were freshly isolated from the Well5-derived osteosarcoma orthotopic lung metastasis model in mice and then total RNA was extracted from sorted cells using Trizol (Invitrogen). Three biological replicates were performed and all samples were subjected to strict quality control. Gene expression profiling was conducted by Shanghai Biotechnology Corporation using Affymetrix U133 plus 2.0 arrays (Affymetrix, Santa Clara, CA). All data were analyzed according to the manufacturer's protocol. Raw data generated from Affymetrix CEL files were normalized by RMA background correction; values were log_2_ transformed. Comparison of the data sets by t test showed that 991 of a total of 28916 probe-sets (3.4%) were differentially expressed (fold changes >2, P <0.05). For the enrichment of P values of each GO term, we used Fisher's exact test to calculate P values and R package stats to calculate FDR (q value) by BH method (www.r-project.org). All microarray data have been deposited with Gene Expression Omnibus (GEO) under accession number GSE85537.

### qRT-PCR analysis

Total RNA from cultured cells was extracted using Trizol reagent (Invitrogen) according to the manufacturer's protocol. qRT-PCR was performed to amplify the cDNA using the SYBR Premix Ex Tag kit(TaKaRa) and an ABI 7500 Sequencing Detection System (AppliedBiosystems, Foster City, CA, USA). The results from qRT-PCR were normalised using the threshold cycle of GAPDH. Targeted genes were amplified with primers listed below: TSP1, 5’-CTCAGGACCCATCTATGATAAAACC-3’ and 5’-AAGAAGGAAGCCAAGGAGAAGTG-3’; FAK, 5’-ACTCATCGAGAGATCGAGATGG-3’ and 5’-GCCCTAGCATTTTCAGTCTTGC-3’; GAPDH, 5’-GGACCTGACCTGCCGTCTAG-3’ and 5’-GTAGCCCAGGATGCCCTTGA-3’.

### Western blot analysis

Whole-cell lysates were prepared using pre-chilled RIPA (50 mM Tris/HCl, pH 7.4, 150 mM NaCl, 1 mM EDTA, 1% Nonidet P-40, 0.1% SDS, 0.5% deoxycholate). The cell lysates were centrifuged at 12,000×g for 20 min at 4°C and the supernatants were collected for protein concentration determination. The total proteins were separated on 10% SDSPAGE gel and blotted onto a nitrocellulose membrane (Millipore). The membrane was incubated with blocking buffer and then was incubated overnight with appropriate primary antibodies at 4 °C. The primary antibodies used were anti-TSP1 (A6.1; Santa Cruz, USA), anti-p-FAK (Y397) (Cell Signaling Technology, USA), anti-FAK (Cell Signaling Technology, USA), anti-MMP2 (Cell Signaling Technology, USA), anti-MMP9 (Cell Signaling Technology, USA), anti-Fibronectin 1(Abcam, Cambridge, UK, USA), and anti-GAPDH (Santa Cruz, USA). Membranes were then washed three times in TBST solution for 15 min each time, and then incubated with secondary antibodies. The membranes were visualized using LI-COR infrared imaging system (LI-COR) following the manufacturer's guidance.

### Immunohistochemistry

Paraffin-embedded tissue sections from osteosarcoma specimens were given a heat pretreatment of 60°C for one hour, then dewaxed in xylene, re-hydrated in an ethanol series (100–50%) and treated in 0.01 mol/L citrate buffer (pH 6.0) for antigen retrieval. After inhibition of endogenous peroxidase activity for 30 min with methanol containing 0.3% H_2_O_2_, the sections were incubated overnight in a moist chamber at 4°C with the primary antibodies anti-TSP1 (A6.1; Santa Cruz, USA). The following experimental procedure was according to the manufacturer's instructions of the LSAB+ kit (Dako, USA). The cytoplasm was counterstained with hematoxylin.

### Wound healing assay

Cells were seeded in six-well plates and cultured until they reached confluence. Wounds were scratched on the monolayer of cells using 20 μL pipette tips. Plates were washed once with fresh medium to remove non adherent cells after the cells had been cultured for 0, 24 or 48 h, and then photographed.

### Migration and invasion assays

For migration assay, 5×10^4^ osteosarcoma cells were suspended in serum-free DMEM and plated on 24-well chambers (Corning Costar, NY, USA) coated without Matrigel. For the invasion assay, the upper chamber was precoated with Matrigel (BD Bioscience, CA, USA) according to the manufacturer's protocols before 5×10^4^ cells in serum-free DMEM were added to the chamber. After 24 hours, the cells that crossed the inserts were stained with 0.5% crystal violet and were counted under an inverted microscope. Values are expressed as mean cell numbers in 5 random fields of view.

### Mouse xenograft

All animal experiments were carried out in accordance with the approved guidelines provided by the Animal Studies Committee of Shanghai Ruijin Hospital. For orthotopic lung metastasis model, human osteosarcoma cells Well5 (with or without TSP1 knockdown) were counted and dilutions of 2×10^6^ viable cells in 100 μl PBS were transplanted orthotopically into the proximal tibia of 6-week-old female severe combined immunodeficient (SCID) mice as previously described [47]. And primary tumor size was measured every week after implantation. At 9 weeks after injection, the mice were then sacrificed and the harvested primary tumors and lungs were fixed in formaldehyde for histopathology analysis (n = 12 for negative control cells and shTSP1 cells, respectively) or observed until their death for the survival curve (n = 12 for negative control cells and shTSP1 cells, respectively).

### Statistical analysis

Results are expressed as mean and standard deviation (SD). Differences between means were studied using a 2-tailed t test or ANOVA when 2 groups or more than 2 groups were compared respectively. The correlation between TSP1 expression and clinic pathological characteristics was assessed by the Pearson χ^2^ test. Statistically Significant results are labeled as follows in all figures: *, P <0.05; **, P < 0.01; ***, P < 0.001. Statistical analysis was done with GraphPad Prism5 (San Diego, CA).

## SUPPLEMENTARY MATERIALS FIGURE


