# Supplementary material for: Thrombospondin-1 promotes cell migration, invasion and lung metastasis of osteosarcoma through FAK dependent pathway
Source: Oncotarget. 2017 Apr 26;8(44):75881–92. doi: 10.18632/oncotarget.17427 (PMC5652671; doi:10.18632/oncotarget.17427)
Supplement: Supplementary file 1 [file oncotarget-08-75881-s001.pdf]

Thrombospondin-1 promotes cell migration, invasion and lung metastasis of osteosarcoma through FAK dependent pathway

SUPPLEMENTARY MATERIALS

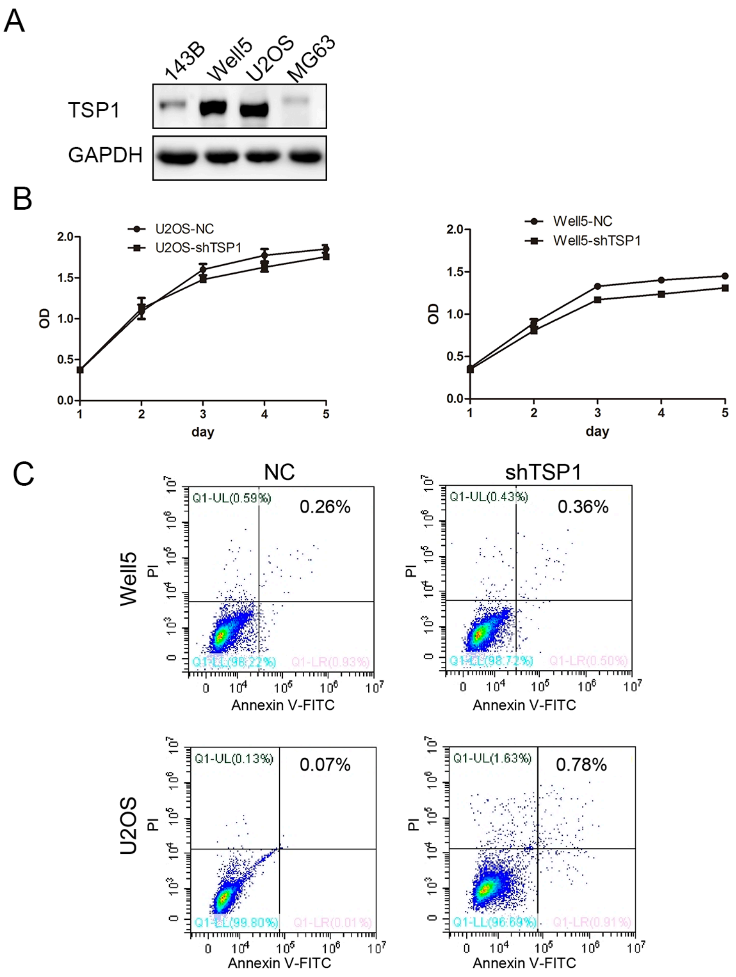

Supplementary Figure 1: (A) Western blot analysis of TSP1 expression in 143B, Well5, U2OS and MG63 cells. **(B)** Silencing TSP1 has no significant effect on the growth of osteosarcoma cells as revealed by CCK-8 assay. Lenti-shTSP1 versus negative control group (NC), P>0.05. Values shown are the mean absorbance {plus minus} SD for five wells from one experiment, and are representations of three independent experiments. **(C)** Lenti-shTSP1 and negative control group (NC) transfected cells were stained with Annexin V-APC and PI for flow cytometric analysis. Shown are representative images of three independent experiments.
